# PADI2 gene confers susceptibility to breast cancer and plays tumorigenic role via ACSL4, BINC3 and CA9 signaling

**DOI:** 10.1186/s12935-016-0335-0

**Published:** 2016-07-29

**Authors:** Huifeng Wang, Bing Xu, Xiaoqian Zhang, Yabing Zheng, Yan Zhao, Xiaotian Chang

**Affiliations:** 1 Medical Research Center of Shandong Provincial Qianfoshan Hospital, Shandong University, Jingshi Road 16766, Jinan, 250014 Shandong People’s Republic of China; 2 Taian City Central Hospital, Longtan Road 29, Taian, 271000 Shandong People’s Republic of China; 3 Clinical Laboratory of PKUCare Luzhong Hospital, Taigong Road 65, Zibo, 250400 Shandong People’s Republic of China

**Keywords:** Peptidylarginine deiminase (PAD), Citrullination, PADI2 (peptidylarginine deiminase isoform 2), ACSL4 (long-chain fatty acyl-CoA synthetase 4), BIRC3 (baculoviral IAP repeat containing 3), CA9 (carbonic anhydrase IX)

## Abstract

**Background:**

Peptidylarginine deiminase (PAD) post-translationally converts arginine residues to citrulline residues. Recent studies have suggested that PADI2 (PAD isoform 2), a member of the PAD family, is involved in the tumorigenic process of some tumors, especially breast cancer. However, little is known about the mechanisms of PADI2 in tumorigenesis. This study aimed to elucidate the tumorigenic role and regulatory pathway of PADI2 in breast tumors.

**Methods:**

The Sequenom MassARRAY and TaqMan genotyping methods were used to investigate the correlation between PADI2 gene SNPs and various tumor risks. PCR array analyses, including cancer pathway finder and signal transduction PCR arrays, were performed to investigate the tumorigenic pathway of PADI2 in the MCF-7 breast cancer cell line following treatment with anti-PADI2 siRNA. Cell proliferation, apoptosis and transwell migration assays were performed to observe the effect of PADI2 in MCF-7 cells treated with anti-PADI2 siRNA.

**Results:**

Both Sequenom MassARRAY and TaqMan genotyping assays demonstrated that SNP rs10788656 in the PADI2 gene was significantly associated with breast cancer. PCR arrays indicated that inhibiting PADI2 expression significantly increased expression of CA9 and decreased expression of ACSL4 and BIRC3 in MCF-7 cells, which was verified using real-time PCR. Inhibiting PADI2 expression also significantly decreased the migration ability of MCF-7 cells but did not affect cell proliferation or apoptosis.

**Conclusions:**

The PADI2 gene confers susceptibility to breast cancer. PADI2 expression contributes to abnormal migration of breast tumor cells. PADI2 affects tumorigenesis in breast tumor cells by regulating the expression of ACSL4, BINC3 and CA9, which are known to promote abnormal lipid metabolism and cell invasion of tumors.

**Electronic supplementary material:**

The online version of this article (doi:10.1186/s12935-016-0335-0) contains supplementary material, which is available to authorized users.

## Background

Peptidylarginine deiminase (PAD) catalyzes the conversion of arginine residues to citrulline residues in the presence of excessive calcium. This enzymatic reaction is referred to as citrullination or, alternatively, deimination. PAD-mediated post-translational citrullination plays important roles in protein function and structural stability and, therefore, significantly affects biochemical pathways by altering the structure and function of the substrates [1, 2]. Five mammalian PAD family members (PAD or PADI 1–4 and 6) are all encoded by a cluster of genes on chromosome 1p36.1 [3]. The pathological roles of the PAD family members and citrullination in pathogenesis are garnering increasing interest [4, 5].

Peptidylarginine deiminase isoform 2 (PAD2/PADI2) has been implicated in cancer. McElwee et al. found that PADI2 expression is regulated by EGF (epidermal growth factor) in mammary cancer cells and appears to play a role in the proliferation of normal mammary epithelium. They furthermore found that PADI2 mRNA expression is highly correlated with HER2 (human epidermal growth factor receptor-2), a well-known diagnostic maker for breast cancer, in a luminal breast cancer cell line [6]. Cherrington et al. also found that EGF up-regulates PADI2 transcription and translation in CMT25 canine mammary tumor cells [7]. In addition, Bhattacharya et al. detected PADI2 expression and citrullination in glaucoma [8], and McElwee et al. recently reported that PADI2 overexpression in transgenic mice promotes spontaneous skin neoplasia [9]. The above studies support a suggestion that PADI2 is involved in the tumorigenic process of some tumors, especially breast cancer [10]. However, little is known about the detailed mechanisms of PADI2 function during the tumorigenic process.

The present study investigated the possible association between candidate SNPs (single nucleotide polymorphisms) in the PADI2 locus and various tumors. We aimed to determine whether these common polymorphisms in the PADI2 region are associated with various tumor risks using the Sequenom MassARRAY genotyping method. The genotyping result was verified using a TaqMan genotyping assay in independent cohorts. Based on the genotyping result, we focused on analyzing the tumorigenic role of PADI2 in cultured tumor cells. We also used a PCR array to investigate the regulatory pathway of PADI2 in tumorigenesis. The PCR array result was verified with real-time PCR.

## Results

### Genotyping SNPs located in the PADI2 locus

Four tag SNPs were genotyped using samples from cohorts of patients with breast cancer, cervical carcinoma, esophageal carcinoma, gastric carcinoma, liver cancer, lung cancer, ovarian cancer and rectal carcinoma and from healthy controls using the Sequenom MassARRAY system. The case–control analysis showed a significant difference in allele frequency and genotype frequency for rs2746533 in PADI2 between gastric carcinoma patients and controls. The analysis also showed a significant difference in allele frequency and genotype frequency for rs2076616 between gastric carcinoma patients and controls. In addition, the analysis showed a significant difference for rs10788656 between the following groups: breast cancer patients and controls in genotype frequency; cervical carcinoma patients and controls in allele frequency; esophageal carcinoma patients and controls in allele frequency and genotype frequency; lung cancer patients and controls in allele frequency; and rectal carcinoma patients and controls in genotype frequency. The above results are shown in Table 1. Rs79395834 did not show single nucleotide polymorphisms in the Chinese population.

**Table 1:** Genotyping result of Sequenom MassARRAY (control n = 760)

SNP identity-gene	Breast cancer n = 629	Cervical carcinoma n = 258	Esophageal carcinoma n = 397	Gastric carcinoma n = 487	Liver cancer n = 191	Lung cancer n = 506	Ovarian cancer n = 107	Rectal carcinoma n = 162
rs2746533-PADI2								
Allele	C T	C T	C T	C T	C T	C T	C T	C T
Case (freq)	5 (0.004) 1251 (0.996)	1 (0.003) 315 (0.997)	2 (0.004) 462 (0.996)	0 (0.000) 974 (1.000)	1 (0.003) 305 (0.997)	3 (0.003) 1003 (0.997)	0 (0.000) 214 (1.000)	3 (0.011) 265 (0.989)
Control (freq)	5 (0.004) 1111 (0.996)	5 (0.004) 1111 (0.996)	5 (0.004) 1111 (0.996)	5 (0.004) 1111 (0.996)	5 (0.004) 1111 (0.996)	5 (0.004) 1111 (0.996)	5 (0.004) 1111 (0.996)	5 (0.004) 1111 (0.996)
Odds ratio (% 95 CI)	0.888090 (0.256423–3.075787)	0.705397 (0.082109–6.060061)	0.961905 (0.185950–4.975867)		0.728525 (0.084793–6.259333)	0.664606 (0.158423–2.788111)		2.515472 (0.597393–10.592010)
Fisher’s p value	0.851382	0.749245	0.963054	0.036536	0.771955	0.573935	0.326631	0.19301
Genotype	C/T T/T	C/T T/T	C/T T/T	C/T T/T	C/T T/T	C/T T/T	C/T T/T	C/T T/T
Case (freq)	5 (0.008) 623 (0.992)	1 (0.006) 157 (0.994)	2 (0.009) 230 (0.991)	0 (0.000) 487 (1.000)	1 (0.007) 152 (0.993)	3 (0.006) 500 (0.994)	0 (0.000) 107 (1.000)	3 (0.022) 131 (0.978)
Control (freq)	5 (0.009) 553 (0.991)	5 (0.009) 553 (0.991)	5 (0.009) 553 (0.991)	5 (0.009) 553 (0.991)	5 (0.009) 553 (0.991)	5 (0.009) 553 (0.991)	5 (0.009) 553 (0.991)	5 (0.009) 553 (0.991)
Odds ratio (% 95 CI)	0.887640 (0.255616–3.082381)	0.704459 (0.081700–6.074199)	0.961739 (0.185248–4.992999)			0.663600 (0.157779–2.791021)		2.532825 (0.597703–10.733088)
Fisher’s p value	0.85107	0.748734	0.962971	0.036311	0.771484	0.573208	0.325716	0.191714
HWE for case (df = 1)	0.920197	0.968156	0.947409	1	0.967636	0.946498	1	0.895718
HWE for control (df = 1)	0.915315	0.915315	0.915315	0.915315	0.915315	0.915315	0.915315	0.915315
rs2076616-PADI2								
Allele	C T	C T	C T	C T	C T	C T	C T	C T
Case (freq)	744 (0.594) 508 (0.406)	242 (0.602) 160 (0.398)	433 (0.568) 329 (0.432)	585 (0.603) 385 (0.397)	187 (0.607) 121 (0.393)	594 (0.590) 412 (0.410)	125 (0.584) 89 (0.416)	195 (0.606) 127 (0.394)
Control (freq)	863 (0.569) 655 (0.431)	863 (0.569) 655 (0.431)	863 (0.569) 655 (0.431)	863 (0.569) 655 (0.431)	863 (0.569) 655 (0.431)	863 (0.569) 655 (0.431)	863 (0.569) 655 (0.431)	863 (0.569) 655 (0.431)
Odds ratio (% 95 CI)	1.111577 (0.955026–1.293791)	1.147958 (0.917529–1.436256)	0.998901 (0.837961–1.190752)	1.153256 (0.978920–1.358640)	1.172970 (0.913311–1.506450)	1.094258 (0.930993–1.286154)	1.065984 (0.797481–1.424888)	1.165363 (0.911687–1.489624)
Fisher’s p value	0.171997	0.227272	0.990214	0.088135	0.21118	0.274552	0.666036	0.221572
Genotype	C/C C/T T/T	C/C C/T T/T	C/C C/T T/T	C/C C/T T/T	C/C C/T T/T	C/C C/T T/T	C/C C/T T/T	C/C C/T T/T
Case (freq)	211 (0.337) 322 (0.514) 93 (0.149)	73 (0.363) 96 (0.478) 32 (0.159)	121 (0.318) 191 (0.501) 69 (0.181)	160 (0.330) 265 (0.546) 60 (0.124)	57 (0.370) 73 (0.474) 24 (0.156)	175 (0.348) 244 (0.485) 84 (0.167)	31 (0.290) 63 (0.589) 13 (0.121)	62 (0.385) 71 (0.441) 28 (0.174)
Control (freq)	246 (0.324) 371 (0.489) 142 (0.187)	246 (0.324) 371 (0.489) 142 (0.187)	246 (0.324) 371 (0.489) 142 (0.187)	246 (0.324) 371 (0.489) 142 (0.187)	246 (0.324) 371 (0.489) 142 (0.187)	246 (0.324) 371 (0.489) 142 (0.187)	246 (0.324) 371 (0.489) 142 (0.187)	246 (0.324) 371 (0.489) 142 (0.187)
Odds ratio (% 95 CI)								
Fisher’s p value	0.163453	0.483706	0.921641	0.009749	0.458051	0.547721	0.107007	0.32765
HWE for case (df = 1)	0.095379	0.962603	0.672532	0.001867	0.937468	0.946205	0.028433	0.329539
HWE for control (df = 1)	0.918961	0.918961	0.918961	0.918961	0.918961	0.918961	0.918961	0.918961
rs10788656-PADI2								
Allele	C G	C G	C G	C G	C G	C G	C G	C G
Case (freq)	1119 (0.894) 133 (0.106)	351 (0.869) 53 (0.131)	655 (0.862) 105 (0.138)	859 (0.886) 111 (0.114)	283 (0.919) 25 (0.081)	888 (0.883) 118 (0.117)	190 (0.888) 24 (0.112)	280 (0.870) 42 (0.130)
Control (freq)	1380 (0.909) 138 (0.091)	1380 (0.909) 138 (0.091)	1380 (0.909) 138 (0.091)	1380 (0.909) 138 (0.091)	1380 (0.909) 138 (0.091)	1380 (0.909) 138 (0.091)	1380 (0.909) 138 (0.091)	1380 (0.909) 138 (0.091)
Odds ratio (% 95 CI)	0.841353 (0.654673–1.081266)	0.662264 (0.472460–0.928320)	0.623810 (0.476051–0.817430)	0.773874 (0.594307–1.007696)	1.132000 (0.725548–1.766147)	0.752542 (0.580357–0.975814)	0.791667 (0.500143–1.253114)	0.666667 (0.461171–0.963731)
Fisher’s p value	0.176816	0.016208	0.000577	0.05659	0.584658	0.031606	0.317835	0.030167
Genotype	C/C C/G G/G	C/C C/G G/G	C/C C/G G/G	C/C C/G G/G	C/C C/G G/G	C/C C/G G/G	C/C C/G G/G	C/C C/G G/G
Case (freq)	494 (0.789) 131 (0.209) 1 (0.002)	152 (0.752) 47 (0.233) 3 (0.015)	279 (0.734) 97 (0.255) 4 (0.011)	382 (0.788) 95 (0.196) 8 (0.016)	129 (0.838) 25 (0.162) 0 (0.000)	394 (0.783) 100 (0.199) 9 (0.018)	84 (0.785) 22 (0.206) 1 (0.009)	122 (0.758) 36 (0.224) 3 (0.019)
Control (freq)	628 (0.827) 124 (0.163) 7 (0.009)	628 (0.827) 124 (0.163) 7 (0.009)	628 (0.827) 124 (0.163) 7 (0.009)	628 (0.827) 124 (0.163) 7 (0.009)	628 (0.827) 124 (0.163) 7 (0.009)	628 (0.827) 124 (0.163) 7 (0.009)	628 (0.827) 124 (0.163) 7 (0.009)	628 (0.827) 124 (0.163) 7 (0.009)
Odds ratio (% 95 CI)								
Fisher’s p value	0.018396	0.052554	0.001004	0.161302	0.487441	0.097391	0.549667	0.098232
HWE for case (df = 1)	0.010705	0.768654	0.161016	0.459996	0.272946	0.370499	0.737085	0.856144
HWE for control (df = 1)	0.749415	0.749415	0.749415	0.749415	0.749415	0.749415	0.749415	0.749415

To verify the above results, genotyping for tag SNP rs10788656 was performed in samples from cohorts of patients with breast cancer, colon cancer, esophageal cancer, cervical cancer, gastric cancer, liver cancer, lung cancer and rectal cancer and from healthy controls using the TaqMan method. Allele frequencies and gene frequencies of the SNP did not deviate from Hardy–Weinberg Equilibrium (HWE) in both cases and controls. The allele frequency (odds ratio 1.607331; 95 % CI [1.072208–2.409527], p = 0.020737) and gene frequency (p = 0.022085) for this SNP demonstrated a statistically significant association with breast cancer. Following multiple-test correction, this SNP still showed a significant difference in allele frequency and genotype frequency in breast cancer. Genotyping did not detect a significant difference in allele or genotype frequencies for rs10788656 between patients with colon cancer, cervical cancer, esophageal cancer, gastric cancer, liver cancer, lung cancer or rectal cancer (p > 0.05).

### Detecting cell proliferation, apoptosis and migration of MCF-7 cells treated with anti-PADI2 siRNA

**Fig. 1:**
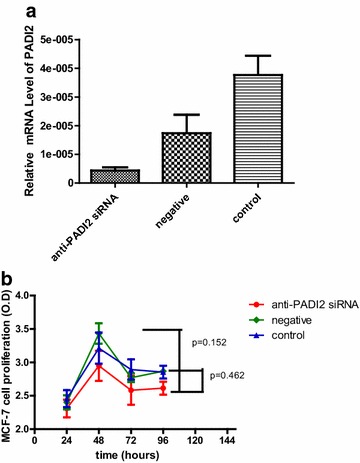
Proliferation of MCF-7 cells that were treated with anti-PADI2 siRNA. **a** Real-time assay detected the PADI2 mRNA level in the anti-PADI2 siRNA-treated cells. **b** CCK-8 assay detected viable cell numbers as represented by an O.D. value at 405 nm. The cells treated with HiPerFect transfection reagent were used as normal controls, and the cells treated with Allstar siRNA were used as negative controls

The proliferation of MCF-7 cells that were treated with anti-PADI2 siRNA was measured using the CCK-8 assay. Real-time assay detected significantly decreased transcription of PADI2 in the anti-PADI2 siRNA treated cells. A significant decline in cell proliferation was not detected in the siRNA-treated MCF-7 cells compared with cells that were treated with the Allstar siRNA (p = 0.152) and cells that were treated with only HiPerFect transfection reagent (p = 0.462). These results, which are shown in Fig. 1, suggest that PAD2 is not involved in regulating cell proliferation.

**Fig. 2:**
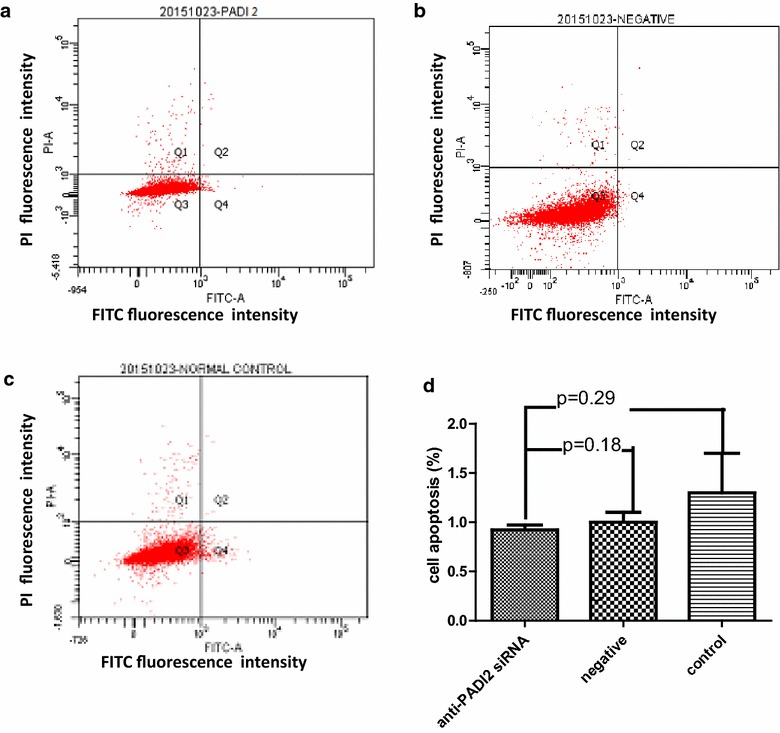
Apoptosis in MCF-7 cells that were treated with anti-PADI2 siRNA, as measured using an annexin V cell apoptosis assay. **a** The cells were treated with anti-PADI2 siRNA. **b** The cells treated with Allstar siRNA were used as negative controls. **c** The cells without siRNA treatment were used as normal controls. **d** The result of the apoptosis assay is shown in a *graph*

The effect of PADI2 on apoptosis in MCF-7 cells was determined using flow cytometric analysis with annexin V/PE and 7-AAD double staining. Compared with the Allstar siRNA treated cells (p = 0.12) and cells treated with HiPerFect transfection reagent (p = 0.18), the number of apoptotic cells was not significantly changed in the anti-PADI2 siRNA-treated cells. The result, shown in Fig. 2, demonstrates that down-regulation of PADI2 expression cannot induce apoptosis in MCF-7 cells.

**Fig. 3:**
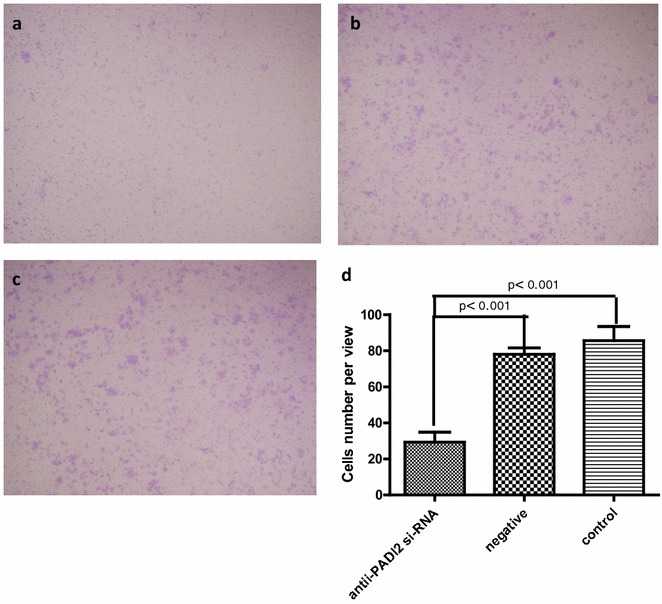
Migration of MCF-7 cells that were treated with anti-PADI2 siRNA, as measured using a transwell migration assay. **a** The cells were treated with anti-PADI2 siRNA. **b** The cells treated with Allstar siRNA were used as negative controls. **c** The cells without siRNA treatment were used as normal controls. **d** The result of the migration measurement is shown in a *graph*. Original magnification: ×4.2

MCF-7 cell migration was examined using a 2-compartment transwell system. A significantly decreased migration of MCF-7 cells was observed when PADI2 expression was suppressed by anti-PADI2 siRNA compared with the cells treated with Allstar siRNA (p < 0.001) or HiPerFect transfection reagent (p < 0.001). The result, shown in Fig. 3, demonstrates that down-regulation of PADI2 expression can inhibit migration of MCF-7 cells.

### Determining the regulatory pathway of PADI2 in MCF-7 cells

**Fig. 4:**
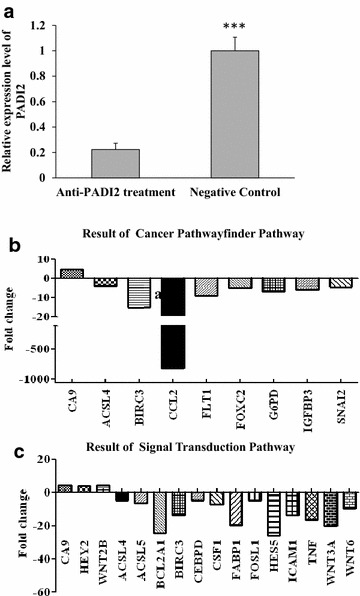
Determination of the regulatory pathway of PADI2 using PCR arrays. The tumor cell line MCF-7 was treated with anti-PADI2 siRNA. Cells treated with Allstar siRNA were used as a negative control. **a** PADI2 expression level was detected using real time-PCR in MCF-7 cells following treatment with anti-PADI2 siRNA. The PADI2 transcription level in the treated cells was normalized with the mRNA level in negative control cells. **b** Cancer pathway finder (**c**) Signal Transduction PCR arrays were used to detect altered expression of tumor-related genes in the treated cells. Fold changes were calculated and expressed as log-normalized ratios of the expression level in siRNA-treated cells/the expression level in negative control. Genes with at least a fourfold change in expression were considered biologically significant

The breast tumor cell line MCF-7 was treated with anti-PADI2 siRNA. The cells treated with Allstar siRNA were used as a negative control. The inhibition of PADI2 expression was confirmed in the cultured tumor cells using real-time PCR. A series of Qiagen PCR arrays were used to examine the changes in gene expression in the tumor cell line and to determine the tumorigenic pathway. The cancer pathway finder PCR array revealed 9 genes, including ACSL4, BIRC3,CA9, CCL2, FLT1, FOXC2, G6PD, IGFBP3 and SNAI2, with significantly altered expression in the siRNA-treated MCF-7 cells. The signal transduction PCR array detected 16 genes, including CA9, HEY2, ACSL4, ACSL5, BCL2A1, BIRC3, CEBPD, CSF1, FABP1, FOSL1, HES5, ICAM1, TNF, WNT3A and WNT6, with significantly altered expression in the treated cells. The results are shown in Fig. 4.

**Fig. 5:**
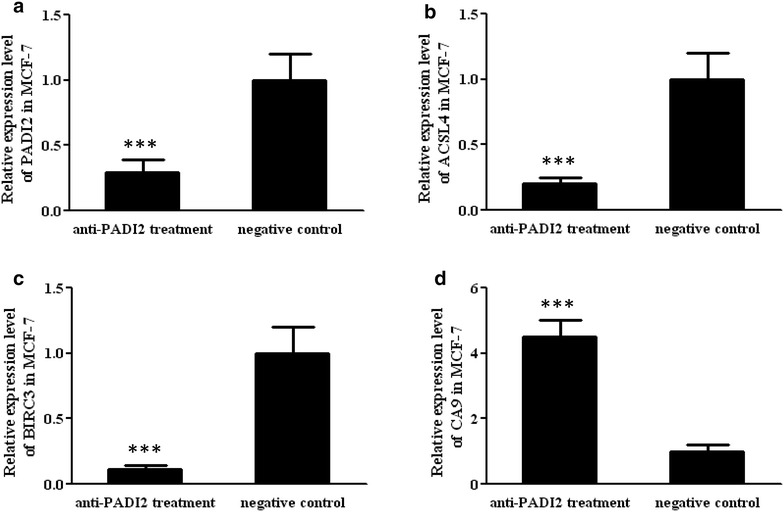
Determination of the mRNA expression levels of PADI2, ACSL4, BICR3 and CA9 in MCF-7 cells using real-time PCR. MCF-7 cells were treated with anti-PADI2 siRNA. Cells treated with Allstar siRNA were used as negative controls. The transcription levels of the target genes in the treated cells were normalized with their mRNA levels in negative control cells. **a** PADI2 expression in MCF-7 cells, **b** ACSL4 expression in MCF-7 cells, **c** BICR3 in MCF-7 cells, **d** CA9 in MCF-7 cells. ***p < 0.001

Real-time PCR was applied to verify the above PCR array analysis results. The increased expression of CA9 and decreased expression of BIRC3 and ACSL4 in MCF-7 cells was detected following treatment with the anti-PADI2 siRNA, which was consistent with the results of the PCR array analysis. These results are shown in Fig. 5. Other genes that were detected with significantly altered expression by PCR array, such as CCL2, were not confirmed by real-time PCR.

## Discussion

In the present study, we used the Sequenom MassARRAY system to genotype the tag SNPs rs2746533, rs79395834, rs2076616 and rs10788656 in the PADI2 locus to determine their association with susceptibility to various tumors. Analysis indicated that the SNPs rs2746533, rs2076616 and rs10788656 had a significant difference in allele frequency, genotype frequency or both between breast cancer, cervical carcinoma, gastric carcinoma, lung cancer and rectal carcinoma cases and the controls. The above genotyping result was verified using TaqMan assays in independent cohorts of various tumors. The analysis showed a significant difference in allele frequency and genotype frequency for rs10788656 between breast cancer samples and the controls, which was completely consistent with the Sequenom MassARRAY result. The two genotyping methods with two independent cohorts provided the first evidence that the PADI2 gene confers susceptibility to breast cancer. We therefore focused on investigating the pathogenic mechanism of PADI2 in breast cancer.

SNP rs10788656/rs58133422 is located in intron 1 of the PADI2-encoding gene and is 6773 bp upstream from exon 2. Thus far, there have been no reports about the bio-function of this SNP. A tag SNP is a representative single nucleotide polymorphism in a region of the genome with high linkage disequilibrium that represents a group of SNPs called a haplotype. It is feasible to genotype a few tag SNPs to determine a potential association with phenotypes without genotyping every SNP in the chromosomal region. We will screen more SNPs in this region, especially in the exon region and its surrounding region, to find functional SNPs and determine how these SNPs affect PADI2 expression and the enzyme activity in which PADI2 has been implicated in some diseases and, more recently, in cancers. Cherrington et al. demonstrated that PADI2 is expressed in canine mammary gland epithelium and that levels of histone citrullination in this tissue correlate with PADI2 expression [10]. In another study, the authors found that normal human and canine mammary epithelia also showed strong cytoplasmic and nuclear expression of PADI2. However, PADI2 expression was reduced in mammary carcinomas from both species. Feline mammary carcinomas had complete loss of nuclear PADI2 expression. The authors suggested that loss of nuclear PADI2 expression may therefore represent a marker of progression towards more aggressive neoplasia [11]. Additionally, McElwee et al. reported that PADI2 expression increases during the transition of normal mammary epithelium to fully malignant breast carcinomas, with a strong peak for PADI2 expression and activity [6]. The authors recently reported that approximately 37 % of transgenic mice overexpressing PADI2 developed spontaneous neoplastic skin lesions. They then found that the human squamous cell carcinoma cell line A431 with overexpressed PADI2 was more tumorigenic and contained elevated levels of markers for inflammation and epithelial-mesenchymal transition [9]. Cherrington et al. found that PADI2 expression is low in anestrus and has extensive expression in the entire epithelium of the mammary duct in late diestrus. EGF up-regulates PADI2 transcription and translation in CMT25 cells, a canine mammary tumor cell line. At the subcellular level, PADI2 is expressed in the cytoplasm, and to a lesser extent, the nucleus of these epithelial cells [7]. In the present study, a significant decline in cell migration was detected in MCF-7 cells when PADI2 transcription was suppressed by siRNA, indicating that PADI2 expression contributes to abnormal migration of breast tumor cells. This result is also in accordance with our finding about the role of the PADI2 gene in the risk of susceptibility to breast cancer. Our studies and others indicate that PADI2 may function as an oncogene to mediate the tumorigenic process.

The tumorigenic pathway of PADI2 was analyzed in the MCF-7 tumor cell line using tumorigenesis-related PCR arrays. RNA interference with PADI2 expression resulted in significant alteration of the expression of some genes involved in tumor progress and signal transduction. Real-time PCR verified the increased expression of CA9 and decreased expression of BIRC3 and ACSL4 in the breast cancer cell line.

Previous studies have shown that key enzymes involved in lipid metabolic pathways are differentially expressed in normal tissues compared with tumor tissues. ACSL4, which mainly esterifies arachidonic acid into arachidonoyl-CoA, is increased in breast, colon and hepatocellular carcinoma. Maloberti et al. reported that ACSL4 is significantly up-regulated in the highly aggressive MDA-MB-231 breast cancer cells and regulates the expression of cyclooxygenase-2 (COX-2) [12]. Orlando et al. reached a similar conclusion [13]. Wu et al. suggested that ACSL4 can serve as both a biomarker for and a mediator of an aggressive breast cancer phenotype [14]. The present study detected decreased expression of ACSL4 and decreased cell migration ability in MCF-7 cells treated with anti-PADI2 siRNA. Combined with the above findings, the present study suggests that increased expression of PADI2 in breast cancer cells may contribute to aggressive activity by stimulating ACSL4 expression and the ACSL4-mediated lipid metabolism signaling pathway.

The BIRC3 gene encodes a member of the IAP (inhibitor of apoptosis proteins) family of proteins that inhibit apoptosis by binding to tumor necrosis factor receptor-associated factors TRAF1 and TRAF2. BIRC3 was down-regulated in response to 1,25D in human mammary epithelial cells [15, 16]. ER and NF-κB can up-regulate the anti-apoptotic activity of BIRC3. NF-κB, acting through two response elements, is required for ER recruitment to an adjacent estrogen response element (ERE) in the BIRC3 promoter [17]. The current study detected decreased expression of BIRC3 in breast cancer cells when PADI2 expression was suppressed using anti-PADI2 siRNA. It is possible that the increased expression of PADI2 in breast cancer cells up-regulates BIRC3 expression through ER and NF-κB to simulate anti-apoptotic functions.

CA9 catalyzes the reversible metabolism of carbon dioxide to carbonic acid and has been linked to malignant transformation and hypoxia in various cancers. CA9 is a powerful marker used to diagnose various types of metastatic cancers, including cervical, renal, breast and head and neck tumors. Preclinical studies in cultured cells have clearly demonstrated that CA9 stimulates the metastatic properties of cancer cells. Expression of the CA9 protein and the BRCA1 (breast cancer 1) protein are inversely correlated in patients with breast cancer. Patients with high levels of CA9 expression show significantly worse overall survival. High CA9 protein expression occurs in patients with the BRCA1 mutant signature and low levels of the BRCA1 protein [18]. Hypoxia-regulated CA9 expression is associated with poor survival in patients with invasive breast cancer [19, 20]. The current study detected increased expression of CA9 in the anti-PADI2 siRNA-treated breast cancer cell lines, supporting the importance of the PADI2-CA9 pathway in breast cancer progression. In addition, some studies have reported that a subgroup of gastric cancers retains CA9 expression in cancer cells at the invasion front and that expression of CA9 is associated with increased invasion [21]. These findings and ours support the hypothesis that CA9 expression mediated by PADI2 may contribute to invasion and advanced tumor progression in breast cancer.

We previously detected increased expression of PADI4 in a variety of malignant tissues and in the blood of tumor patients [22, 23]. We recently detected citrullination of α-enolase, heat shock protein 60, cytokeratin 8 and tubulin beta in ECA, H292, HeLa, HEPG2, Lovo, MCF-7, PANC-1, SGC and SKOV3 tumor cell lines using proteomic methods [24]. Zhang et al. found that stimulation of ER α-positive cells with 17 β-estradiol (E2) promotes citrullination of histone H3 arginine 26 (H3R26) on chromatin. They further found that citrullination of H3R26 is catalyzed by PADI2, whereas H4R3 is catalyzed by PADI4. They suggested that estrogen stimulation induces the recruitment of PADI2 to target promoters by ERα, whereby PADI2 then catalyzes H3R26, which leads to local chromatin decondensation and transcriptional activation [25]. It is possible that both PADI2 and PADI4 are expressed in breast tumor tissues and function by different pathogenic pathways.

This study potentially had intrinsic methodology limitations. Some genes that may play important roles in tumorigenesis are not included in the PCR arrays. In addition, genes with at least a fourfold change in expression were considered biologically significant in the study, based on the instructions of the manufacturer. Thus, some important genes that are related to breast tumorigenesis may not have been analyzed in our study. In addition, we used cultured cell lines to investigate tumorigenic mechanisms. The observed mechanisms may not be completely similar to the mechanisms in tumor tissues.

## Conclusions

The present study demonstrated that PADI2 significantly increases susceptibility to breast cancer. PADI2 expression contributes to migration of breast tumor cells. Moreover, the study found that PADI2 can regulate ACSL4, BINC3 and CA9 expression to advance abnormal lipid metabolism and cell invasion in breast tumors. These findings may be useful for further understanding the tumorigenic process.

## Methods

### Tissue collection

Blood samples were collected in the Clinical Laboratory of Qilushihua General Hospital (Zibo, Shandong, China), a branch hospital of Shandong Provincial Qianfoshan Hospital. The tumor diagnosis was verified by histological methods, and pathological categorization was performed according to the World Health Organization (WHO) classification system. All patients signed informed consent forms, and the study was approved by the ethics committee of Shandong Provincial Qianfoshan Hospital.

### Genomic DNA isolation and Sequenom MassARRAY genotyping

Peripheral blood samples were collected from patients with breast cancer (n = 629, aged 25–73 years, mean 47.6 years), cervical carcinoma (n = 258, aged 25–78 years, mean 50 years), esophageal carcinoma (n = 397, 159 female, aged 41–81 years, mean 60 years), gastric carcinoma (n = 487, 217 female, aged 21–84 years, mean 56.9 years), liver cancer (n = 191, 35 female, aged 25–86 years, mean 54.2 years), lung cancer (n = 506, 151 female, aged 25–88 years, mean 59 years), ovarian cancer (n = 107, aged 19–78 years, mean 51.7 years) and rectal carcinoma (n = 162, 68 female, aged 21–79 years, mean 55.2 years). A total of 760 (442 female, aged 17–87 years) healthy individuals with a mean age of 44.8 years donated blood. Blood samples were put into Monovette tubes containing 3.8 % sodium citrate. Genomic DNA was extracted from whole blood samples with the Omega E-Z 96 Blood DNA kit (Omega, USA) according to the manufacturer’s protocol.

Tag single nucleotide polymorphisms (tag SNPs) across the PADI2 locus were identified by searching the HapMap database. Only SNPs with a minor allele frequency (MAF) greater than 5 % and a pair-wise r^2^ ≥ 0.8 were considered. Four tag SNPs, including rs2746533, rs79395834, rs2076616 and rs10788656, in the PADI2-encoding gene were selected and genotyped using an allele-specific MALDI-TOF mass spectrometry assay (Sequenom MassARRAY). The polymorphism spanning fragments were amplified by PCR. Primers for the amplification and extension reactions were designed using MassARRAY Assay Design Version 3.1 software (Sequenom, San Diego, CA). Genotyping was then performed using the Sequenom MassARRAY iPLEX platform.

### TaqMan genotyping

To verify the above genotyping result, tag SNP rs10788656 was selected for genotyping in new cohorts of patients with breast cancer (n = 285, 285 women, mean age = 47.65), colon cancer (n = 144, 55 women, mean age = 54.13), esophageal cancer (n = 285, 40 women, mean age = 61.20), cervical cancer (n = 190, 190 women, mean age = 52.75), liver cancer (n = 190, 42 women, mean age = 54.05), lung cancer (n = 190, 56 women, mean age = 58.17), gastric cancer (n = 190, 44 women, mean age = 56.97), and rectal cancer (n = 136, 50 women, mean age = 54.61), as well as in healthy controls (n = 285, 71 women, mean age = 40.1).

Genomic DNA was extracted as described above. The genomic DNA was diluted to a final concentration of 15–20 ng/μl for the genotyping assays. The assays were run on a ViiA 7 DX (Life Technology) and evaluated according to the manufacturer’s instructions. Reactions were carried out in a total volume of 10 μl using the following amplification protocol: denaturation at 95 °C for 10 min, 50 cycles of denaturation at 95 °C for 15 s and then annealing and extension at 60 °C for 1 min. The genotype of each sample was determined by measuring allele-specific fluorescence using the TaqMan Genotyper software V1.2 (Life Technology). Duplicate samples and negative controls were included to check the accuracy of genotyping.

Genotyping quality was examined by a detailed quality control procedure consisting of a >95 % successful call rate, duplicate calling of the genotypes, internal positive control samples and HWE testing. The SNPs were analyzed for association by comparing the MAF between the cases and controls. Dominant and recessive models were considered with respect to the minor allele. The association of the SNPs with diseases was evaluated using odds ratios (OR) with 95 % confidence intervals (CI). Fisher’s exact test was used for comparisons between categorical variables. p values less than 0.05 were considered statistically significant. Genotypic association was assessed using Plink v1.07 (http://pngu.mgh.harvard.edu/purcell/plink/) and SHEsis (http://analysis.bio-x.cn/myAnalysis.php) software [26, 27]. Bonferroni single-step correction was performed by Plink v1.07.

### Cell culture and siRNA interference

The MCF-7 breast cancer cell line was cultured in Dulbecco’s modified Eagle’s medium (DMEM) supplemented with 10 % fetal calf serum, 50 U/mL penicillin and 50 μg/mL streptomycin in an atmosphere of 5 % CO_2_ at 37 °C. SiRNA oligonucleotides targeting the PADI2 gene (target sequence: 5′ CCCGTTCTTCGGCCAACGCTA 3′) were designed and synthesized by QIAGEN (Germany). The cultured tumor cells were transfected with siRNA at 20 nM using the HiPerFect transfection reagent (QIAGEN) according to the manufacturer’s protocol. The cells were harvested for analysis 48 h after transfection. Parallel experiments with Allstar siRNA were used as a negative control. Inhibition of PADI2 expression in the cell line was verified using real-time PCR.

### PCR array analysis

Total RNA was isolated from the anti-PADI2 siRNA-treated MCF-7 cells using Trizol solution (Invitrogen, USA) according to the manufacturer’s protocol. PCR arrays are sets of optimized real-time PCR primer assays in 96-well plates and are used to monitor the expression of genes related to a disease state or pathway. In this study, the Cancer PathwayFinder PCR array and Signal Transduction Pathway PCR array (Qiagen) were used to identify the pathogenic pathways of PADI2 in tumorigenesis. The array layout is shown in Additional file 1. The PCR array analysis was conducted using a ViiA7 DX instrument (Life Science) according to the manufacturer’s instructions. The procedure begins with the conversion of experimental RNA samples into first-strand cDNA using the RT^2^ First Strand Kit. Next, the cDNA is mixed with an appropriate RT^2^ SYBR Green Mastermix. This mixture is aliquoted into the wells of the RT^2^ Profiler PCR Array. PCR is performed, and the relative expression level is determined using the data from the real-time cycler and the ∆∆CT method. The raw array data were processed and analyzed by the PCR array data analysis system at http://sabiosciences.com/pcrarraydataanalysis.php. Fold changes were calculated and expressed as log-normalized ratios of the siRNA treated cells/controls. Based on the instructions of the manufacturer, genes with at least a fourfold change in expression were considered biologically significant in the study.

### Real-time PCR

Total RNA was extracted from tumor tissues or MCF-7 cells and then reverse-transcribed in a final volume of 10 μL using the RNA PCR Kit (TaKaRa, Japan). Real-time PCR was conducted using a ViiA7 DX instrument according to the manufacturer’s protocol. The reactions were completed in a total volume of 10 µL, containing 1 µL of cDNA, 5 µl of SYBR Green Real-time PCR Master Mix (ToYoBo, Japan) and 1 µL of each primer. PCR amplification cycles were performed using the following conditions: 10 s at 95 °C, 40 cycles of 5 s at 95 °C and 31 s at 60 °C. For each sample, two reactions were performed at the same time. One reaction was performed to determine the mRNA level of the target gene and the other to determine the β-actin level. PCR products were confirmed by melt curve analysis. Relative mRNA expression was calculated using the comparative threshold cycle (Ct) method. The relative target gene expression level was normalized to the β-actin mRNA expression level. Forward primer and reverse primer sequences for the amplification were as follows: PADI2: 5′ TGAAAGAGGTGAAGAACCTTG 3′ and 5′ GTTTAGGTACTGGAAGCAGAC 3′; β-actin: 5′-TGGCACCCAGCACAATGAA-3′ and 5′-CTAAGTCATAGTCCGCCTAGAAGCA-3′; ACSL4 (long-chain fatty acyl-CoA synthetase 4): 5′-CATAGCAATTTGATAGCTGGAA-3′ and 5′-TATCCAATCCTGCAGCCAT-3′; BIRC3 (baculoviral IAP repeat containing 3): 5′-GGTAACAGTGATGATGTCAAATG-3′ and 5′-TAACTGGCTTGAACTTGACG-3′; CA9 (carbonic anhydrase IX): 5′-GCCTTTCTGGAGGAGGG-3′ and 5′-AGATATGTCCAGTCCTGGG-3′.

### Cell proliferation assay

MCF-7 cells were seeded into 96-well culture plates and incubated until they reached 80 % confluence. The culture was then treated with anti-PADI2 siRNA and incubated for 24–72 h at 37 °C in 5 % CO_2_. Following the addition of 10 µl of Cell Counting Kit-8 (CCK-8, Dojindo) solution to each well, the cells were incubated for an additional 4 h. Absorbance was measured at 450 nm with a spectrophotometer (Spectramax 190; Molecular Devices). Growth curves were generated from the average values of five wells per group.

### Cell apoptosis assay

Apoptosis in the siRNA-treated MCF-7 cells was analyzed using flow cytometry (FACSAria II, BD Biosciences). The cultured cells were washed twice with PBS and were resuspended in binding buffer at a concentration of 1 × 10^6^ cells/mL. The cell suspensions (1 × 10^5^ cells/100 µL) were transferred into 5-mL culture tubes, and 5 µL of annexin V-phycoerythrin (eBioscience) and 5 µL of 7-amino-actinomycin (eBioscience) were then added. The cells were gently vortexed and incubated at room temperature in the dark for 15 min. Subsequently, another 400-µL aliquot of binding buffer was added. Flow cytometry was performed within 4 h of staining.

### Transwell migration assay

Transwell inserts (8.0-µm pore size) with a polycarbonate filter (Costar ^®^) were used to examine the effects of PADI2 on cell migration. Anti-PADI2 siRNA-treated MCF-7 cells (5 × 10^4^ cells/200 µL) were suspended in serum-free media and added to the upper chamber, and 500 µL of complete DMEM media was added to the lower chamber. Following incubation for 24 h, the filter was immersed in methanol for 15 min at room temperature and then was treated with 0.25 % crystal violet stain for 10 min at room temperature prior to washing with water. The cells that had migrated to the lower side of the filter were counted with an inverted fluorescence microscope.

### Statistical analysis

Data were analyzed using the two-tailed Student’s t test. Differences were considered significant at p < 0.05. Experiments were performed with triplicate samples and were performed three times or more to verify the results.

## Additional file

10.1186/s12935-016-0335-0 Determination of the pathogenic pathway of PADI2 using PCR arrays. The array layout shows tumor-related genes in (**A**) Cancer pathway finder and (**B**) Signal transduction PCR arrays.
