# Supplementary figures and images for: PADI2 gene confers susceptibility to breast cancer and plays tumorigenic role via ACSL4, BINC3 and CA9 signaling
Source: Cancer Cell Int. 2016 Jul 29;16:61. doi: 10.1186/s12935-016-0335-0 (PMC4966586; doi:10.1186/s12935-016-0335-0)

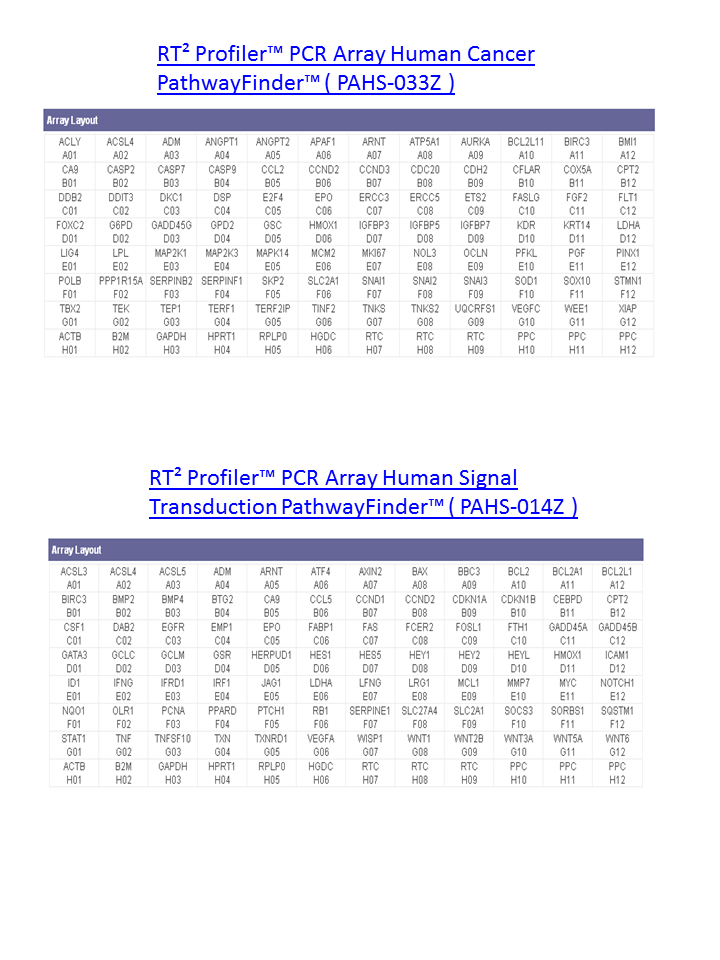

Supplement: Supplementary file 1 — 10.1186/s12935-016-0335-0 Determination of the pathogenic pathway of PADI2 using PCR arrays. The array layout shows tumor-related genes in (A) Cancer pathway finder and (B) Signal transduction PCR arrays. [file 12935_2016_335_MOESM1_ESM.tif]
